# β-Thalassemia minor & renal tubular dysfunction: is there any association?

**DOI:** 10.1186/s12882-021-02602-9

**Published:** 2021-12-07

**Authors:** Mohsen Vakili Sadeghi, Maryam Mirghorbani, Roghayeh Akbari

**Affiliations:** 1 Social Determinants of Health Research Center, Health Research Institute, Babol University of Medical Sciences, Babol, Iran; 2 Students Research Committee, Babol University of Medical Sciences, Babol, IR Iran; 3 Department of Internal Medicine, Ayatollah Rouhani Hospital, Keshavarz Boulevard, Babol, Mazandaran Iran

**Keywords:** β-Thalassemia minor, Renal tubular dysfunction, Fraction excretion, Sodium, Uric acid

## Abstract

**Objective:**

Beta(β)-thalassemia is one of the most common hereditary hematologic disorders. Patients with thalassemia minor (TM) are often asymptomatic and the rate of renal dysfunction is unknown in these patients. Due to the high prevalence of renal dysfunction in Iran, the current study aimed to determine renal tubular dysfunction in patients with beta-TM.

**Methods:**

In this case-control study, 40 patients with TM and 20 healthy subjects were enrolled and urinary and blood biochemical analysis was done on their samples. Renal tubular function indices were determined and compared in both groups. Data was analyzed by SPSS software, version 20.0.

**Results:**

The fraction excretion (FE) of uric acid was 8.31 ± 3.98% in the case and 6.2 ± 34.71% in the control group (*p* = 0.048). Also, FE of potassium was significantly higher in patients with TM (3.22 ± 3.13 vs. 1.91 ± 0.81; *p* = 0.036). The mean Plasma NGAL level was 133.78 ± 120.28 ng/mL in patients with thalassemia and 84.55 ± 45.50 ng/mL in the control group (*p* = 0.083). At least one parameter of tubular dysfunction was found in 45% of patients with thalassemia.

**Conclusion:**

Based on the results of this study, the prevalence of tubular dysfunction in beta-thalassemia minor patients is high. Due to the lack of knowledge of patients about this disorder, periodic evaluation of renal function in TM patients can prevent renal failure by early diagnosis.

## Introduction

β-thalassemia is one of the most common hereditary hematologic disorders, characterized by disturbances in beta chain hemoglobin synthesis [1]. It is the most common single-gene disorder in Iran and more than 25,000 major β-thalassemia have been reported [2]. Three main clinical forms of β-thalassemia include thalassemia major, thalassemia intermedia (TI) and (TM) [3]. Patients with TM or Cooley’s anemia require regular transfusions before age 24 months to survive. Thalassemia intermedia patients do not require or rarely require blood transfusions. They may be asymptomatic up to adulthood but their sign and symptoms include biliary gallstone, jaundice, osteoporosis, hepatosplenomegaly and mass lesions related to extramedullary hematopoiesis. Individuals with TM or thalassemia carries have mild anemia with no or minimal symptoms [4, 5]. Clinical manifestations of beta thalassemia result from one or both beta globin gene mutations. Patients with TM are homozygotes or double heterozygotes, TI results from heterozygotes or homozygote and individuals with TM are heterozygote for beta gene mutations [6].

Thalassemia can be sub grouped to transfusion dependent thalassemia (TDT) and non-transfusion dependent thalassemia (NTDT). Phenotype of NTDT includes heterogeneous thalassemia genotypes that do not require frequent transfusions but is more severe than TM and have several complications due to ineffective erythropoiesis, extramedullary hematopoiesis and iron overload. Five form of NTDT has been described: beta thalassemia intermedia, hemoglobin E β-thalassemia, HbH disease, hemoglobin S β-thalassemia and hemoglobin C β thalassemia [7].

The improved TM and TI survival has allowed previously unrecognized renal complications to emerge [8]. The effect of thalassemia on the kidney has not been extensively evaluated. Up to 60% of patients with beta-thalassemia major have been reported to develop signs of tubular dysfunction [9].

Renal dysfunction is an uncommon complication in patients with β-thalassemia [10]. Mild impairment of tubular function and decreased glomerular filtration rate (GFR) have been reported in older patients with alpha-thalassemia, β-thalassemia major and hemoglobin E/β-thalassemia [1, 3]. Tubular dysfunction among patients with beta-thalassemia has been related to long-term anemia with chronic hypoxemia, intravascular and extravascular hemolysis, chronic blood transfusions, iron overload, as well as desferrioxamine toxicity [8, 11, 12]. Regular screening of renal function in high-risk thalassemia patients with diabetes, hypertension, proteinuria and GFR < 60 mL/min/1.73 m^2^ and elderly people in order to detect early renal involvement and thus prevent the onset of renal impairment is recommended [13].

Although there are many available data about renal involvement in patients with beta-thalassemia major, the changes in renal functions of TM were reported less [9, 14–18]. For a long time, there have been no reports regarding renal impairment in these patients, until 2002, which Oktenli and Bulucu, for the first time reported a 20-year-old case of TM in Turkey, who was investigated due to positive Glucosuria with dip-stick analysis. The tests reported a 24-h urine glucose secretion of 5 g and tubular proteinuria, a sign of his involvement in renal tubular dysfunction [17]. Patients with TM may have a lower frequency of hyperuricosuria and phosphaturia [1]. The most common probable cause of renal tubular dysfunction in thalassemia intermediate is iron deposition in the epithelial cells of the tubules. Another factor may be hypoxia due to hemolysis of red blood cells and their shorter life span and this hypoxia has the greatest effect on the adrenal cortical epithelial cells that are more susceptible to oxygen deficiency [16, 17]. In these patients, tubular and glomerular dysfunction depends on the severity of anemia, frequency of blood transfusion, and iron load [19]. Considering the possibility of renal dysfunction in patients with TM and the high prevalence of this type of thalassemia in Mazandaran province, this study aimed to determine renal tubular dysfunction in these patients.

## Materials and methods

In this case-control study, patients referring to the hematology clinic of Rouhani Hospital of Babol between March 2017 to March 2018 because of anemia were evaluated. Inclusion criteria were confirmation of β-thalassemia minor with hemoglobin electrophoresis and patients’ satisfaction to participate in the study. Patients with cardiovascular disease (cardiac arrhythmia, aortic stenosis, ischemic heart disease, hypertension, and heart failure), liver disease, any type of cancer, active infection, history of renal disease (proteinuria, increased creatinine, acute kidney injury history), diabetes, drug addiction, taking medications that are excreted through the kidneys within the last 3 months and pregnancy were excluded from the study.

Forty β-thalassemia minor patients were selected by the simple randomization selection method, serum ferritin, complete blood count and hemoglobin electrophoresis were performed for all patients. Beta thalassemia minor was confirmed by complete blood count, HbA2 ≥ 3.5 g/dL and HbF < 5 g/dL in hemoglobin electrophoresis. For eligible persons, the research plan was fully explained and written informed consent was obtained. Also, 20 healthy staffs and physicians of the Rohani hospital, without any history of chronic diseases, were selected in the control group by the simple randomization selection method. After recording the demographic data, a complete history of drug use and underlying diseases was obtained. Then, 5 cc venous blood sample and 24-h urine were obtained from both groups.

Urine specimens were evaluated for appearance, pH, specific gravity, sodium, potassium, phosphorus, calcium, uric acid, creatinine, microalbumin and the presence of ketones, glucose, protein, bilirubin, urobilinogen, red and white blood cells, bacteria, casts, crystals, mucus and epithelial cells. Blood samples were also analyzed for fasting blood sugar (FBS), blood urea nitrogen (BUN), creatinine, uric acid, sodium, potassium, calcium and phosphorus. Urinary Neutrophil Gelatinase Associated Lipocalin (NGAL) was also measured.

Sodium and potassium were measured using flame photometer (Assel Co., Rome, Italy), urine microalbumin and biochemical tests were measured using conventional commercial kits and spectrophotometric method. Also, for biochemical analysis and measuring urinary NGAL, Human NGAL Rapid ELISA Kit (KIT037) manufactured by the Bioporto diagnostic company, Denmark was used by using the monoclonal ELISA sandwich method.

Calculation of renal function indices was based on the following: Glucosuria: Positive glucose in the urine; Hypercalciuria: Calcium > 300 mg in men and >  250 mg in women in 24-h urine; Hyperphosphaturia: Phosphate > 1000 mg in 24-h urine; Uricosuria: uric acid > 750 mg in 24-h urine; Microalbuminuria: Microalbumin > 30 mg in 24-h urine.

GFR was calculated based on the Modification of Diet in Renal Disease (MDRD) formula [20]:

GFR = 186.3 × (Plasma Cr)-1.154 × (age)-0.203 (For women, it was multiplied with 0.742).

$$\mathrm{FENa}=\frac{\mathrm{urinary}\ \mathrm{Na}\ \left[\mathrm{mg}/\mathrm{mL}\right]\ }{\mathrm{serum}\ \mathrm{Na}\ \left[\mathrm{mg}/\mathrm{mL}\right]} \times \frac{\mathrm{serum}\ \mathrm{creatinine}\ \left[\mathrm{mg}/\mathrm{mL}\right]}{\mathrm{urinary}\ \mathrm{creatinine}\ \left[\mathrm{mg}/\mathrm{mL}\right]}\times 100$$

Normal values of FENa were less than 1%.

$$\mathrm{FEK}=\frac{\mathrm{urinary}\ \mathrm{K}\ \left[\mathrm{mg}/\mathrm{mL}\right]\ }{\mathrm{serum}\ \mathrm{K}\ \left[\mathrm{mg}/\mathrm{mL}\right]} \times \frac{\mathrm{serum}\ \mathrm{creatinine}\ \left[\mathrm{mg}/\mathrm{mL}\right]}{\mathrm{urinary}\ \mathrm{creatinine}\ \left[\mathrm{mg}/\mathrm{mL}\right]}\times 100$$

Normal values of FEK were less than 15%.

$$\mathrm{FEUA}=\frac{\mathrm{urinary}\ \mathrm{uric}\ \mathrm{acid}\ \left[\mathrm{mg}/\mathrm{mL}\right]\ }{\mathrm{serum}\ \mathrm{uric}\ \mathrm{acid}\ \left[\mathrm{mg}/\mathrm{mL}\right]} \times \frac{\mathrm{serum}\ \mathrm{creatinine}\ \left[\mathrm{mg}/\mathrm{mL}\right]}{\mathrm{urinary}\ \mathrm{creatinine}\ \left[\mathrm{mg}/\mathrm{mL}\right]}\times 100$$

Normal values of FEUA were < 10%.

Renal Tubular Reabsorption of Phosphate (TmP/GFR)=.

$$\mathrm{Plasma}\ \mathrm{Phosphate}=\frac{\mathrm{Plasma}\ \mathrm{Creatinin}\mathrm{e}\times \mathrm{Urine}\ \mathrm{phosphate}\ }{\mathrm{Urine}\ \mathrm{Creatinin}}$$

TmP/GFR values < 2.88 were considered to be normal.

Data was analysed using Statistical Package for statistical analysis (SPSS) version 20.0. Information description was by frequency tables and related charts. To characterize qualitative characteristics, frequency and percentage were used, and for the quantitative characteristics, the mean and range of variations were used. Qualitative data was analyzed using chi-square and Fisher exact tests and quantitative variables by t-test and Mann-Whitney tests for comparison of averages. The statistical significance level in all tests was considered 0.05, so that *P*-value < 0.05 showed a significant statistical difference.

The research protocol was approved by the Ethics Committee of Babol University of Medical Sciences (Registration code = MUBABOL.REC.1395.150) and all methods were carried out in accordance with relevant guidelines and regulations and the study was conducted in accordance with the Declaration of Helsinki.

## Results

The mean age of the case and control groups were 41.93 ± 17.71 and 39.20 ± 10.13 years, respectively (*p* = 0.527). In the study population, 37.5% of cases (15 of 40 cases) and 45% (9 of 20 cases) of controls were male (*p* = 0.576). The mean hemoglobin, hematocrit, MCV, MCH and MCHC were significantly lower in the case group compared to the controls (*p* = 0.0001) but no significant difference was found between the two groups for white blood cells, red blood cells and platelets (*p* > 0.05) (Table 1).

**Table 1:** Comparison of biochemical parameters and fractional excretion of different parameters in the two groups

Parameters	Case *n* = 40	Control *n* = 20	*P* value
WBC (× 10^3^)	6.94 ± 2.11	7.087 ± 14.16	0.782
RBC (× 10^6^)	4.91 ± 0.77	5.02 ± 0.54	0.411
Hemoglobin	10.53 ± 1.07	14.10 ± 1.37	< 0.0001
Hematocrit	33.33 ± 3.08	41.36 ± 3.35	< 0.0001
MCV	65.09 ± 6.03	86.91 ± 3.62	< 0.0001
MCH	21.02 ± 3.15	29.60 ± 1.74	< 0.0001
MCHC	32.12 ± 2.93	34.05 ± 1.16	0.001
Platlet (×10^3^)	315.22 ± 12.56	268.95 ± 72.62	0.134
FE_Na_	0.27 ± 0.14	0.21 ± 0.15	0.099
FE_Ca_	1.04 ± 0.73	0.84 ± 0.76	0.227
FE_K_	3.22 ± 3.13	1.91 ± 0.81	0.036
FE_UA_	8.31 ± 3.98	6.34 ± 2.71	0.048

The mean plasma concentrations of creatinine, uric acid, phosphorus, sodium and potassium were not significantly different in the two groups, but BUN and calcium were significantly lower in patients with thalassemia (*p* = 0.007 and *p* = 0.002, respectively). Also, urine microalbumin, creatinine, uric acid, phosphorus, calcium, sodium and potassium were not significantly different between the two groups (*p* > 0.05).

In the case group, glucosuria occurred in 3 patients (7.5%) and proteinuria in 2 patients (5%), while they were not reported in any of the control subjects. There was a significant difference between FE_UA_ in both groups (*p* = 0.048). Also, FE_K_ was significantly higher in patients with TM (*p* = 0.036) but FE_Na_ and FE_Ca_ did not differ significantly (*p* = 0.099 and *p* = 0.227, respectively) (Table 2).

**Table 2:** Comparison of renal tubular dysfunction parameters in two groups

	Case group N (%)	Control group N (%)	*P* Value
Glucosuria	3 (7.5)	0(0)	0.045
Hypercalciuria	4(10)	4(20)	0.422
Hyperphosphaturua	7(17.5)	2(10)	0.704
Microalbuminuria	4(10)	0(0)	0.0001
Uricosuria	8(20)	2(10)	0.471
FE_NA_ > 1%	4(10)	1(5)	0.482
FE_Ca_ > 1%	8(20)	3(15)	0.736
FE_UA_ > 10%	5(12.5)	2(10)	0.776
FE_K_ > 15%	2(5)	0(0)	*NS
TMP/GFR < 2.88	9(22.5)	3(15)	0.734

*NS not significant

The mean eGFR in case and control groups were 89.95 ± 17.55 and 87.93 ± 15.49, respectively (*p* = 0.676). The maximum ratio of TMP/GFR in patients with TM was 0.33 ± 1.03 mg/dL and in normal subjects it was 3.37 ± 0.78 mg/dL, which was not significantly different between the two groups (*p* = 0.193). The mean plasma NGAL in the case and control groups were 133.78 ± 120.28 ng/mL and 84.55 ± 45.55 ng/mL, respectively (*p* = 0.083) (Table 1).

In the comparison of tubular dysfunction factors, glucosuria and microalbuminuria were significantly higher in the thalassemia group (*p* = 0.045 and *p* = 0.0001. respectively). Also, plasma NGAL values > 179 ng/mL was reported in 6 patients with thalassemia (15%) and 1 healthy person (5%), which was significantly higher in patients with thalassemia (*p* = 0.025).

None of the renal tubular dysfunction parameters were seen in 80% of controls and 55% of patients with TM (*p* = 0.046) (Table 3). The mean plasma NGAL in the case group which had microalbuminuria was higher than those without microalbuminuria (*p* = 0.047). Also, NGAL was higher in patients with FENa> 1% and FEUA> 10% than other thalassemic patients (*p* = 0.011 and *p* = 0.004, respectively) (Table 4).

**Table 3:** Comparing the mean NGAL based on renal tubular dysfunction factors in patients with thalassemia

Renal tubular dysfunction factors		Number	mean ± SD	*P* Value
Glucosuria	+	3	127.12 ± 92.13	0.795
	–	37	130.81 ± 102.45	
Hypercalciuria	+	4	150.75 ± 109.57	0.948
	–	36	143.64 ± 123.45	
Hyperphosphaturia	+	7	165.71 ± 146.18	0.446
	–	33	127.01 ± 115.56	
Microalbuminuria	+	4	187.25 ± 120.87	0.047
	–	36	127.83 ± 107.71	
Hypercalciuria	+	8	132.25 ± 107.25	0.969
	–	32	134.16 ± 124.91	
FE_NA_	> 1%	4	137.36 ± 125.41	0.011
	≤1%	36	102.61 ± 55.05	
FE_Ca_	> 1%	8	76.88 ± 49.51	0.137
	≤1%	32	148.02 ± 128.85	
FE_UA_	> 10%	5	273.09 ± 163.81	0.004
	≤10%	35	113.89 ± 100.89	
FE_K_	> 15%	2	76.50 ± 26.16	0.497
	≤15%	38	136.79 ± 122.65	
TMP/GFR	> 2.88%	9	169.11 ± 163.96	0.323
	≤2.88%	31	123.52 ± 105.61

**Table 4:** Comparison of increased NGAL based on the number of renal tubular dysfunction parameters in patients with thalassemia

	NGAL mean ± SD	Normal NGAL N (%)	Elevated NGAL N (%)
No parameters	123.77 ± 108.41	22(100)	0(0)
1–2 parameters	122.50 ± 120.36	4(66.7)	2(33.3)
> 2 parameters	157.75 ± 146.21	8(66.7)	4(33.3)

The mean hemoglobin level in thalassemic patients with NGAL > 176 ng/mL was 10.31 ± 1.73 g/dL and in patients with NGAL < 176 ng/mL, it was 11.93 ± 2.88 g/dL (*p* = 0.052).

The mean plasma NGAL in patients with TM with at least one parameter of renal tubular dysfunction was 146.54 ± 115.59 ng/mL and in patients with no abnormalities in renal tubular function, it was 123.12 ± 77.14 ng/mL (*p* = 0.046) (Table 4). Thalassemic patients were classified into 3 types based on renal tubular dysfunction. The mean NGAL of the third group was significantly higher than the first and second groups (*p* = 0.004 and *p* = 0.025, respectively). The abnormal NGAL was not significantly associated with the number of renal tubular dysfunction parameters (*p* = 0.581).

## Discussion

The association between serum and urinary levels of NGAL and renal dysfunction was evaluated [21–23], but to our knowledge, no biomarker study has been conducted in patients with beta-thalassemia minor so far and this study is the first evaluation in this type.

The results of this study showed evidence of renal tubular disorder in patients with TM and in 45% of patients, at least one parameter of renal tubular dysfunction was reported. Glucosuria and microalbuminuria were significantly higher in thalassemic patients than in healthy subjects, but other parameters of renal tubular damage did not differ in two groups. Also, fraction excretion of uric acid and potassium was significantly higher in patients with thalassemia. For the first time, Oktenli and Bulucu reported glucosuria and tubular proteinuria in a male with β-thalassemia minor [17]. Prabahar and colleagues reported evidence of nephrocalcinosis associated with renal tubular dysfunction, such as hypercalciuria, decreased phosphorus tubular reuptake, hypomagnesemia, and urinary magnesium loss in a 24-year-old woman with TM [18]. Cetin et al. found renal tubular dysfunction as a common complication in patients with TM and 14.6% of these patients had renal tubulopathy symptoms such as hypercalciuria, decreased TRP with hypophosphatemia, hypomagnesemia associated with renal magnesium loss, hypouricemia along with renal excretion of uric acid and tubular proteinuria [14], but no evidence of tubular dysfunction was found in Kalman et al.’s study in patients with TM [16]. In the study of Hoseinzadeh et al. in Shiraz, of 86 patients with TM, 24% had renal tubular dysfunction [15]. Sadeghi-Bojd and colleagues reported symptoms of tubulopathy such as proteinuria, urinary excretion of microglobulin, calciuria, phosphaturia, and uricosuria in thalassemic patients. In their study, creatinine clearance, uric acid and potassium excretion, and tubular reabsorption of phosphorus in patients with TM were significantly different from the control group, but no other tubule injury parameters were different between the two groups [9].

In our study, eGFR was not significantly different between the two groups. In previous studies, GFR did not change significantly in any type of thalassemia (major, intermediate, and minor) [1, 24, 25]. In the study of Nickavar et al., eGFR was insignificantly increased in thalassemic patients, which was probably secondary to anemia and decrease in systemic vascular resistance in addition to elevated renal plasma flow [1]. Both tubular dysfunction and renal glomerular disorder may occur in patients with thalassemia. These disorders are probably due to decreased production of adenosine triphosphatase, oxidative stress, lipid peroxidation, prostaglandin secretion imbalance, hyperdynamic cardiovascular system, increased renal blood flow and glomerular hyperfiltration [13, 25, 26]. Proximal tubular disorder has been reported in 13–60% of patients with all types of thalassemia. Increased urinary excretion of sodium, calcium, phosphorus, magnesium, uric acid, N-acetyl glucosamine, beta 2-microglobulin, retinol binding protein, and glucose in association with decreased urinary osmolality are symptoms of renal tubular dysfunction in thalassemic patients [9, 12]. Increased protein excretion is one of the most common clinical manifestations of renal involvement, which is seen in about 70% of patients with thalassemia with renal impairment [12, 27].

It has been suggested that renal tubular disorder may be secondary to anemia [14]. Changing the cell function due to reducing the oxygen supply to renal tubular cells can be a major cause of this phenomenon. Empirical evidence has shown that anemia, can lead to renal hypoxia [28]. The proximal tubular disorder has been reported in patients with iron deficiency anemia in Özçay et al.’s study [29]. Therefore, tubular iron loading or red blood cell hemolysis toxins are a potential cause of impaired proximal tubular function. In addition, increased iron exchange due to mild hemolysis of microcytic erythrocytes with a significant increase in LDH level may be another factor associated with proximal tubular injury in patients with TM [14]. Therefore, tubular iron or toxins loading due to red blood cell hemolysis is a potential cause of impaired proximal tubular function.

Studies on the function of all patients with TM are very rare. Renal tubular dysfunction in adults with TM can be due to hemolysis, reduced lifespan of erythrocytes, tubular Iron deposit, oxidative lipid peroxidation, and toxins produced from erythrocytes [14, 30]. Tubular dysfunction has also been reported in patients with iron deficiency anemia [29]. But we believe the mechanisms described for tubular injury in TM are not logically acceptable. TM is not usually associated with iron overload. They do not have chronic hemolysis or severe anemia that explains tissue hypoxemia.

Since the renal disease is common and is progressing to the advanced stages silently, screening for renal disease is essential and in case of lack of screening and intervention, the patients suffer from a range of symptoms from non-clinical signs of renal damage to death. On the other hand, creatinine is a non-susceptible marker. So, in this study, NGAL biomarker was used to investigate the probability of tubular injury in patients with TM. In our study, although a difference was found between NGAL in healthy and TM patients, but it did not reach statistical significance. However, elevated plasma NGAL (> 179 ng/mL) was observed in patients with TM. Also, the mean plasma NGAL was significantly higher in TM patients with at least one renal parameters. NGAL is a 21 kilodalton protein of the lipocalin family and is a biomarker for acute renal damage, but is also a new diagnostic tool for the chronic renal disease that reflects the continuous damage of tubular cells in the kidney [31]. This protein is normally released in small quantities by different cells outside the kidney. Therefore, the origin of NGAL production in patients with chronic renal disease is still controversial, and the effect of its tubular origin in comparison with the external production level has not yet been determined [32]. NGAL has been shown to be associated with morphological changes and albuminuria in patients with primary renal disease [33].

There are some limitations to this study. The main limitation of the study was the lack of performing some tests of renal dysfunction, such as urine electrophoresis and N-acetyl-beta-D-glucosaminidase (GcnA), a marker of renal tubular dysfunction. Also, we did not adapt urine materials to diet because the excretion fraction of urinary salt can change based on diet. A cohort study in thalassemic patients and evaluating the changes in renal tubular and glomerular activity over a period of time is recommended.

## Conclusion

According to the results of this study, renal tubular dysfunction is prevalent in patients with TM. Also, we found that increased plasma NGAL level can be considered as the beginning of renal tubular injury in patients with TM. Laboratory measurements of the renal function in these patients at certain time points can prevent further complications. Regarding the prevalence of renal tubular dysfunction in patients with TM and unawareness of the patients about it, a periodic evaluation of renal function can prevent progression of renal dysfunction by identifying them early.

## Data Availability

The trial data used to support the findings of this study are available from the corresponding author upon request.
